# Supplementary material for: A whole-animal phenotypic drug screen identifies suppressors of atherogenic lipoproteins
Source: eLife. 2026 Aug 27;14:RP105314. doi: 10.7554/eLife.105314 (PMC13521566; doi:10.7554/eLife.105314)
Supplement: Supplementary file 2 [file elife-105314-code1.zip › JHDL_primary_result/Thonzonium bromide/Thonzonium bromide_JHDL_26_038_018_2019_11_08_10_43.pdf]

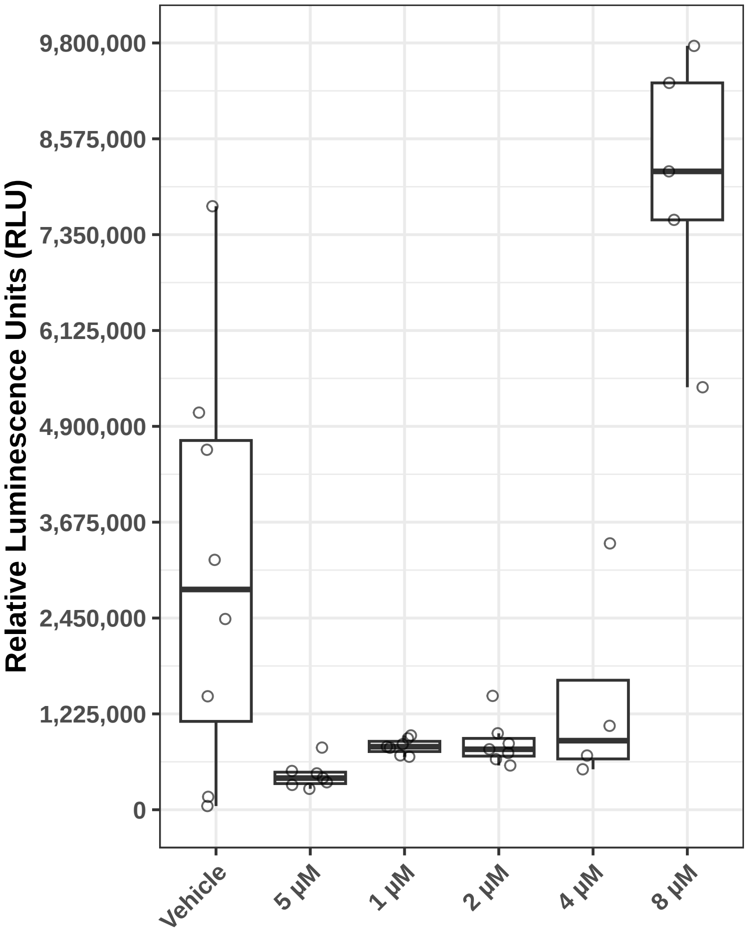

Relative Luminescence Units (RLU)
9,800,000
8,575,000
7,350,000
6,125,000
4,900,000
3,675,000
2,450,000
1,225,000
0
Vehicle
5 μM
1 μM
2 μM
4 μM
8 μM
